# Integrating digital health and remote monitoring: emerging trends in cardiac rehabilitation research for chronic heart failure

**DOI:** 10.3389/fcvm.2026.1774181

**Published:** 2026-04-09

**Authors:** Chun-mei Gao, Ping Fu, Wei Du, Yong Li

**Affiliations:** 1 Department of Rehabilitation Medicine, The Second Affiliated Hospital of Mudanjiang Medical University, Mudanjiang, China; 2 Department of Geriatric Medicine, The Second Affiliated Hospital of Mudanjiang Medical University, Mudanjiang, China; 3 Department of Paediatrics, The Second Affiliated Hospital of Mudanjiang Medical University, Mudanjiang, China; 4 Department of Medical Records, Hongqi Hospital Affiliated to Mudanjiang Medical University, Mudanjiang, China

**Keywords:** cardiac rehabilitation, chronic heart failure, digital health, personalized care, remote monitoring, telemedicine

## Abstract

Chronic heart failure (CHF) represents a major global public health burden. Traditional cardiac rehabilitation models face limitations due to geographical barriers, scheduling conflicts, and high dropout rates, leading to low participation and poor access. This perspective article argues that the deep integration of digital health and remote monitoring represents not merely a technical fix but a paradigm shift toward personalized, continuous, and preventive care. This approach employs a multi-layered framework that combines remote assessment, interactive intervention, and intelligent decision support. It has the potential to increase rehabilitation accessibility and improve patient adherence. However, its impact on long-term clinical effectiveness is context-dependent and varies across patient populations and intervention models. Future progress should rest on three pillars: generating high-quality real-world evidence of long-term value; advancing technology integration and clinical workflow redesign; and bridging the digital divide to ensure health equity. Realizing a patient-centered, intelligent home-based rehabilitation system requires interdisciplinary collaboration and implementation. This is essential for improving long-term outcomes and quality of life for the broad heart failure population.

## 1 Introduction

Chronic heart failure (CHF), constitutes a major global public health burden (1). Its high prevalence, frequent readmissions, and significant mortality rate exert sustained pressure on healthcare systems (1). Robust evidence confirms that comprehensive cardiac rehabilitation—incorporating exercise training, patient education, risk factor management, and psychosocial support—improves cardiac function, exercise capacity, and quality of life while reducing hospital readmissions and mortality in CHF patients (2–4). However, the widespread implementation and long-term effectiveness of cardiac rehabilitation in practice face persistent challenges.

Traditional cardiac rehabilitation typically follows a centralized, in-person, group-based model. This model has inherent limitations: (1) Geographic and accessibility barriers, as frequent travel to specialized centers is difficult for remote, mobility-impaired, or transport-limited patients (5); (2) Time and economic burdens, since regular attendance conflicts with work and incurs costs (6); (3) Physical limitations, as symptoms like fatigue and dyspnea hinder travel and exercise for many with moderate-to-severe CHF (7); (4) Insufficient personalization and high dropout rates, due to limited monitoring and feedback, which reduces engagement, adherence, and program completion (8).

Meanwhile, advances in digital health technologies present opportunities to address these limitations. Digital health encompasses computing platforms, connectivity, software, and sensors for improving health (9). Core components include telemedicine (for remote clinical communication), wearables and biosensors (for continuous passive monitoring of heart rate, rhythm, oxygenation, and activity), mobile health apps (for education, rehab planning, medication reminders, and symptom logging), and artificial intelligence technology (for data integration and analysis) (9–11). Together, they enable a scalable, personalized, and sustainable disease management ecosystem.

This perspective article was developed through a structured narrative synthesis of literature on digital health–enabled cardiac rehabilitation for CHF. A literature search was conducted in PubMed and Web of Science for publications from 2010 to November 2025, using key terms such as “chronic heart failure,” “cardiac rehabilitation,” “digital health,” “telemedicine,” “remote monitoring,” and “telerehabilitation.” Only English-language articles were included. We prioritized high-impact evidence, including clinical guidelines, randomized controlled trials, large observational studies, systematic reviews, and consensus statements relevant to digital or remote rehabilitation in CHF. Studies without relevance to rehabilitation or clinical outcomes were excluded. Given the perspective nature of this study, no formal risk-of-bias assessment or meta-analysis was conducted. This methodological approach facilitates a balanced interpretation of current evidence while identifying key knowledge gaps and implementation challenges.

In contrast to prior narrative reviews, which primarily catalog digital tools or compare remote and center-based models, this perspective offers three distinctive contributions. First, it conceptualizes digital cardiac rehabilitation as a closed-loop, workflow-integrated system rather than a mere collection of technologies, with a proposed three-layer architecture explicitly aligned with clinical decision-making, risk reassessment, and governance logic. Second, it introduces a risk-stratified implementation framework that defines clear eligibility criteria, specifies program assignment pathways—including remote, hybrid, and center-based options—and outlines corresponding gradients of supervision intensity, thereby moving beyond the assumption that remote rehabilitation is universally applicable. Third, the model embeds safety governance and escalation protocols as core structural components by delineating adverse event categories, physiological alert tiers, and clinician accountability pathways, thereby advancing from conceptual endorsement toward operational feasibility. Together, these contributions aim to bridge the gap between technological enthusiasm and clinically actionable implementation.

This perspective article therefore aims to scrutinize the value of integrating digital health and remote monitoring into CHF cardiac rehabilitation; examine current evidence on its effectiveness, safety, and cost-effectiveness; analyze implementation challenges like standardization, data security, liability, the digital divide, and healthcare system integration; and suggest future research directions. The goal is to offer a clear, forward-looking perspective to guide clinical research and practice.

## 2 The integrated framework of digital health and remote monitoring: core components and synergistic value

**Figure 1:**
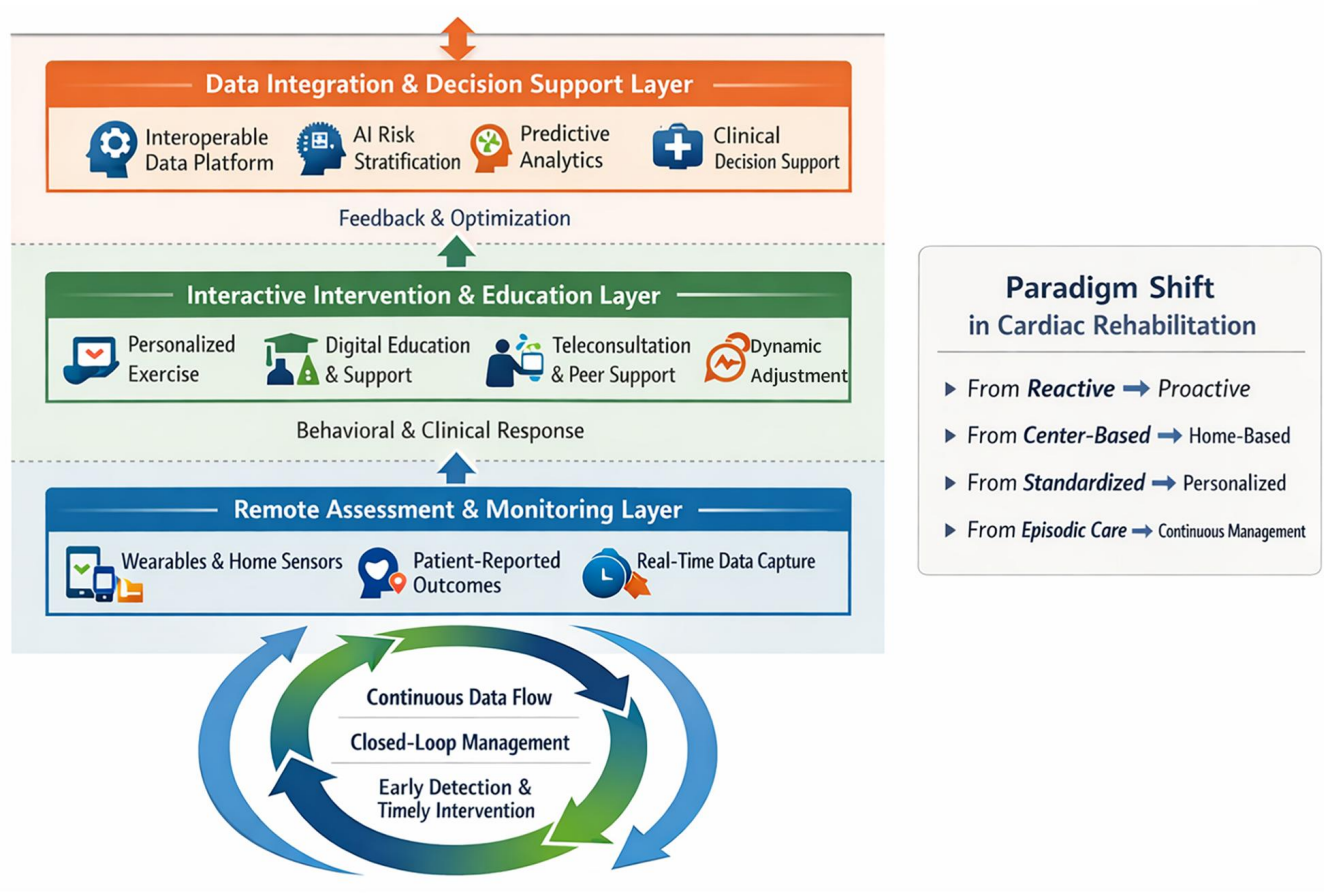
Integrated digital health and remote monitoring framework for cardiac rehabilitation in chronic heart failure.

An integrated framework that combines digital health and remote monitoring technologies offers a solution to the temporal, spatial, and resource constraints of traditional cardiac rehabilitation for CHF (Figure 1). Its core value lies in creating synergistic, multi-level interventions. This requires recognizing that “digital health” should not be treated as a singular intervention, but rather as a collection of distinct technologies. These include remote monitoring devices, mobile health applications, artificial intelligence-based decision support tools, and regulated digital therapeutics. Rather than a simple combination of technologies, this framework is a patient-centered, closed-loop management system. Its effectiveness depends on the nuanced integration of these components, as each differs in clinical validation, regulatory status, legal accountability, and reimbursement pathways. Consequently, the framework is fundamentally data-driven and clinically supported, designed to deliver continuous, personalized, and proactive rehabilitation. Clear differentiation among the incorporated technologies is therefore necessary to avoid conceptual ambiguity and to ensure that the framework for clinical practice and health system planning remains relevant and actionable.

### 2.1 Remote assessment and monitoring layer

This layer provides the foundational monitoring for the framework, enabling continuous and objective patient assessment. First, wearable devices and home monitors facilitate the ongoing collection of core physiological parameters, such as heart rate, rhythm, blood pressure, oxygen saturation, and daily weight (12–14). Rapid short-term weight gain, in particular, is an early and sensitive indicator of fluid retention and impending heart failure decompensation. Second, mobile applications or portals collect patient-reported outcomes—including symptoms like dyspnea and fatigue, quality-of-life scores, and medication adherence (15). This process supplements objective data with crucial subjective and behavioral information. Finally, preset rules or machine learning algorithms analyze this multi-source data in real time (11). They automatically identify abnormal patterns and generate risk alerts to prompt early clinical intervention. This shifts the management paradigm from reactive response to proactive prevention. Therefore, baseline risk stratification and continuous reassessment are critical. They ensure that monitoring intensity and intervention strategies stay aligned with each patient's evolving risk profile.

### 2.2 Interactive intervention and education layer

This layer forms the intervention core, translating assessment data into personalized rehabilitation actions. For exercise, the system can deliver and dynamically adjust safe, individualized prescriptions using video guidance, animations, and wearable device integration (16). These adjustments are based on baseline assessments and real-time physiological feedback. For patient education, it uses interactive multimedia, push notifications, and contextual information to deliver structured instruction on medication, diet, fluid intake, and symptom recognition (17, 18). This approach enhances knowledge retention and promotes behavior change. Furthermore, by incorporating secure video consultations and medically moderated peer-support communities, this layer provides flexible professional guidance and sustained psychosocial support (19). This helps mitigate patient isolation and strengthens long-term engagement.

### 2.3 Data integration and decision support layer

This layer acts as the intelligent hub of the framework, aiming to unlock data's potential for optimizing clinical decisions. A secure, interoperable data platform forms its foundation. This platform integrates multidimensional data—physiological, behavioral, and environmental—from other layers to create a unified patient health profile (20). Leveraging this foundation, artificial intelligence and machine learning enable deeper analysis. This includes: (1) dynamic risk stratification to identify currently stable patients at high risk for future events (21); (2) predicting individual patients' responses to different interventions to inform precise therapy selection (22); and (3) analyzing large-scale anonymized data to identify best practices, thereby comprehensively optimizing protocols and alert thresholds (23).

Despite their potential, artificial intelligence–based systems should be regarded as advanced clinical decision-support tools rather than autonomous decision-makers (24). Their outputs require careful contextualization within the broader clinical picture. Challenges pertaining to algorithm transparency, interpretability, and the risk of automation bias necessitate robust clinician oversight (25, 26). This ensures that algorithmic recommendations are critically evaluated and that final rehabilitation decisions remain guided by comprehensive clinical judgment, safeguarding medico-legal accountability (27). Consequently, this layer is designed not to replace but to augment clinical expertise. It relieves clinicians of labor-intensive data processing and empowers them to make more efficient, evidence-informed, and insight-driven clinical decisions.

In summary, the close collaboration within this three-tiered architecture facilitates a paradigm shift: from passive monitoring to active management, from standardized to personalized care, and from experience-based to data-driven decision-making. It thereby establishes a robust technological foundation for improving the accessibility, adherence, and overall efficacy of cardiac rehabilitation in CHF.

## 3 Current clinical evidence: from non-inferiority verification to value exploration

The evidence base for digital health and remote monitoring in CHF cardiac rehabilitation is evolving. The focus is shifting from validating basic efficacy to exploring comprehensive clinical value and optimal implementation models. The key structural differences between traditional center-based and digitally enabled cardiac rehabilitation models are summarized in Table 1.

**Table 1:** Comparison between traditional center-based and digitally enabled CR models.

Dimension	Traditional center-based CR	Digitally enabled/remote CR
Care setting	Hospital-or clinic-based, in-person	Home-based or hybrid, remotely supported
Accessibility	Limited by location and scheduling	Improved access across time and location
Monitoring strategy	Periodic, visit-based	Continuous or frequent remote monitoring
Data type	Mainly physiological and clinician-reported	Physiological plus patient-reported data
Personalization	Largely standardized programs	Individualized, data-adaptive programs
Patient engagement	Declines over time	Supported by digital feedback tools
Clinical responsiveness	Visit-triggered, reactive	Data-driven, early intervention
Resource utilization	Facility- and staff-intensive	More scalable resource use
Cost considerations	Higher indirect patient costs	Potential system and patient savings
Equity challenges	Barriers for remote patients	Digital divide requiring mitigation

CR, cardiac rehabilitation.

### 3.1 Consensus on efficacy: non-inferiority and potential advantages

Recent randomized controlled trials and meta-analyses increasingly find that digitally-supported home-based cardiac rehabilitation is generally non-inferior to conventional center-based programs for improving key outcomes (28). Specifically, digital and remote models demonstrate comparable effectiveness in improving exercise capacity and health-related quality of life. Meta-analyses report modest but clinical meaningful improvements, alongside improvements in standardized quality of life scores (29, 30). However, evidence on the reduction of all-cause or heart failure–related readmissions remains inconsistent. Furthermore, consistent benefits on mortality or major cardiovascular events have not been clearly demonstrated, especially in studies with short-term follow-up (29, 30). This potential benefit likely arises because remote models overcome geographical and time barriers, improving patient participation and long-term adherence (28). However, most evidence originates from studies involving stable, low-to-moderate risk outpatients (28).

Importantly, current evidence supports the use of digital or remote cardiac rehabilitation mainly for clinically stable patients with low-to-moderate risk heart failure (31). Patients with advanced disease, frequent recent decompensation, multiple comorbidities, significant cognitive impairment, or limited self-management ability may not be suitable for fully remote programs (31). For these individuals, a hybrid model that combines remote rehabilitation with periodic in-person assessments and supervision may offer a safer and more feasible option (32). These findings underscore the need for clear eligibility criteria and ongoing risk evaluation, rather than assuming that remote rehabilitation is appropriate for all patients (33).

### 3.2 Exploration of new models: optimizing pathways and precision management

As the field matures, the research focus has shifted from simple comparisons with traditional care to exploring optimized implementation pathways and strategies for specific patient groups. First, the hybrid model, combining center-based and remote sessions, is widely regarded as a feasible and practical transitional approach (32, 34). It integrates periodic in-person assessments and education with routine remote monitoring and home exercise. This approach maintains safety and necessary human contact while maximizing accessibility and continuity. Second, research increasingly targets the “timing” and “intensity” of intervention (35). A key focus is intensive remote management during the high-risk “vulnerable period” immediately after hospital discharge. The goal is to initiate intensive remote monitoring, medication adjustment, and daily guidance upon discharge to utilize this critical window for preventing early readmission. Third, a significant research direction involves developing and validating integrated digital programs for CHF patients with common comorbidities, such as chronic obstructive pulmonary disease or diabetes (36). These programs aim to coordinate the treatment, monitoring, and education for multiple conditions via a unified platform, thereby improving overall management efficiency.

### 3.3 Evidence limitations: short-term focus and knowledge gaps

Despite promising prospects, the current evidence has notable limitations. First, most studies have short follow-up periods (typically 3–6 months) and report intermediate outcomes (37). There is a lack of robust data on long-term prognostic impact, including effects on mortality and major cardiovascular events. Second, the rapid pace of technological change means evidence based on specific devices or apps can quickly become outdated (38). This poses a challenge for developing stable, long-term clinical guidelines. Finally, from a health economics perspective, while remote models may lower some patient and societal costs, comprehensive cost-effectiveness analyses are still lacking (39). These analyses should include technology procurement, maintenance, training, and data security costs and require support from large-scale, long-term studies. Therefore, future research should prioritize well-designed trials with longer follow-up, a focus on hard clinical endpoints, adaptability to technological change, and thorough evaluation of economic value across different healthcare systems.

### 3.4 Neutral and negative findings: interpreting inconsistent evidence

Despite promising outcomes, certain trials and reviews have reported neutral or mixed results. Some studies found no significant reduction in mortality or major cardiovascular events, particularly in cases with short follow-up periods or interventions based solely on low-intensity monitoring (40). Effectiveness also tends to be lower in older patients with advanced heart failure, significant comorbidities, or limited digital literacy. Collectively, these neutral and negative findings indicate that digital cardiac rehabilitation is not universally effective (41). Its clinical value depends more on appropriate patient selection, tailored intervention intensity, and comprehensive integration into established, multidisciplinary care pathways than on technology deployment alone (10, 41). These observations underscore that the success of digital rehabilitation is contingent upon a deliberate, patient-centered implementation strategy rather than the mere adoption of digital tools. To further clarify how specific intervention components may influence distinct clinical outcomes, prior evidence can be stratified according to key “intervention ingredients,” including personalization intensity, coaching frequency, monitoring modalities, alert design, patient phase, and follow-up duration (Supplementary Table S1). This stratified mapping clarifies that clinical outcomes are not driven by “digitalization” *per se*, but by specific combinations of personalization, supervision intensity, monitoring scope, and patient selection.

## 4 Moving towards clinical practice and scalability: key challenges and mitigation strategies

While digital health and remote monitoring technologies offer a promising approach to expanding cardiac rehabilitation, their real-world feasibility and scalability depend heavily on context. Beyond technical performance, successful implementation requires attention to data reliability, cost-effectiveness, sustained patient engagement, and health system preparedness (42–44). Without addressing these factors, digital rehabilitation may remain limited to pilot studies rather than becoming integrated into routine clinical practice.

### 4.1 Technological challenges: standards, security, and reliability

A robust technological infrastructure is essential for effective integration, yet three major obstacles remain. First, the absence of interoperability standards impedes seamless data exchange between devices, platforms, and protocols from different manufacturers (45). This fragmentation creates data silos that hinder comprehensive analysis and limit clinical utility. Second, ensuring data security and patient privacy throughout the data lifecycle—from transmission and storage to processing—is critical (46). This requires full compliance with regulations like General Data Protection Regulation and Health Insurance Portability and Accountability Act and the use of encryption and anonymization technologies. Third, concerns exist regarding insufficient sensor accuracy and clinical validation (47). Data from consumer-grade wearables need rigorous validation to ensure clinical reliability. This process is necessary to confirm that generated alerts and trends support, rather than hinder, clinical decisions and to avoid alert fatigue or misinterpretation.

From a clinical perspective, data reliability is essential for the safe and effective use of remote monitoring systems. While wearable devices and home sensors support frequent data collection, their accuracy and consistency differ widely depending on device type, brand, and real-world conditions (48). Factors such as motion artifacts, irregular use, and poor signal quality can lead to false alerts or obscure true clinical decline (49, 50). It is also important to note that many consumer-grade devices have not been rigorously validated against clinically accepted reference methods, which reduces their reliability for automated clinical decisions (51). These challenges highlight the need for standardized validation protocols, clinical supervision, and careful interpretation of algorithm-derived results in everyday practice (52).

### 4.2 Clinical and operational challenges: workflow, accountability, and reimbursement

Successful integration hinges on transforming clinical workflows. The core challenge involves redesigning workflows and adapting the roles of clinical teams—including physicians, nurses, and rehabilitation therapists (53). These professionals should become proficient with digital tools, remote communication, and data analysis. From an operational standpoint, continuous remote monitoring can significantly increase workload. The high volume of incoming data demands organized review procedures, while poorly adjusted alert systems may lead to alert fatigue (54, 55). Successful implementation thus relies on redesigning workflows—such as introducing tiered alerts, delegating tasks among multidisciplinary teams, and incorporating decision-support tools to highlight clinically relevant information (56, 57). Some monitoring and interactive tasks may be delegated to trained support staff or to artificial intelligence systems (58, 59). Crucially, without sufficient staffing, training, and institutional support, remote monitoring may add to clinicians' burdens rather than enhance efficiency. This shift requires clearly defined accountability frameworks and updated quality monitoring systems to delineate responsibilities for remote clinical decisions, technical failures, and data interpretation. Furthermore, a significant practical barrier is the lag in reimbursement mechanisms (60). Most healthcare systems lack unified evaluation standards and specific billing codes for digital therapeutics and remote rehabilitation. This gap hinders sustainable service delivery and reduces institutional incentives for adoption.

Moreover, comprehensive economic feasibility remains an unresolved issue. While remote rehabilitation may reduce certain indirect costs, such as travel expenses and lost productivity, robust cost-effectiveness analyses are still limited (61). Substantial technology-related expenditures—encompassing initial device procurement, ongoing software maintenance, cybersecurity infrastructure, staff training, and data management systems—may offset these potential savings, particularly in resource-limited settings (62). The cost-effectiveness profile is also likely to vary significantly across different healthcare systems due to disparities in reimbursement policies, labor costs, and baseline utilization of rehabilitation services (63). Consequently, definitive promotion of digital rehabilitation as a cost-saving strategy awaits evidence from robust, long-term economic evaluations (61).

### 4.3 Patient and societal challenges: equity, engagement, and adaptability

Ensuring equitable access and long-term effectiveness requires addressing deeper patient- and societal-level issues. A primary challenge is the digital divide, which disproportionately affects older adults, low-income populations, and those with limited digital literacy (64). This divide creates barriers in device access, internet connectivity, and operational skills, potentially exacerbating health disparities (65). Addressing this requires simplified technology, community support, and tiered service design. Beyond technological access, the usability and adherence to digital rehabilitation programs are also significantly shaped by clinical characteristics prevalent among patients with CHF (66). These include frailty, cognitive impairment, sensory deficits, and dependence on caregiver assistance (67). Such factors can directly constrain a patient's ability to follow exercise protocols, interpret digital feedback, or respond to system alerts (68). Consequently, without intentionally tailored adaptations and integrated caregiver support, digital rehabilitation may inadvertently favor younger, healthier, and more technologically adept individuals, thus potentially widening existing health inequities.

Another challenge is sustaining long-term patient engagement and maintaining behavioral changes (69). While digital tools can improve short-term engagement through novelty effects and real-time feedback, participation often declines over time as active supervision diminishes (70). Factors such as digital fatigue, limited perceived benefit, and competing life priorities may reduce sustained engagement, indicating that technology alone cannot guarantee long-term adherence (71). To move beyond initial engagement, interventions should integrate behavioral science principles—such as personalized feedback, gamification, and social support networks—and be supplemented by behavioral support strategies, periodic human contact, and adaptive program designs that respond to patients' changing needs to encourage lasting habit formation. Finally, considerations of cultural adaptability and health literacy are essential (72). Educational materials, intervention recommendations, and communication methods should be tailored to patients' cultural contexts, health beliefs, and comprehension levels to ensure effective delivery and acceptance of care.

Importantly, insights can be gained from successful cardiac rehabilitation models used in other cardiovascular conditions. For example, structured rehabilitation programs for patients with atrial fibrillation have shown improvements in functional capacity, symptom management, and quality of life through multidisciplinary support and personalized exercise plans (73, 74). These examples demonstrate that positive outcomes are possible when rehabilitation is integrated into clear clinical pathways, supported by professional guidance, and tailored to individual patient risks (75). These experiences illustrate that successful digital or hybrid rehabilitation depends less on technology itself and more on its integration into clearly defined clinical workflows supported by professional oversight (73). Such evidence supports the potential of digital and hybrid rehabilitation approaches, while underscoring that thoughtful program design—not merely the use of technology—is essential for success.

### 4.4 Safety governance and adverse event management in remote cardiac rehabilitation

Ensuring patient safety is paramount for remote, exercise-based cardiac rehabilitation in CHF. Effective safety governance requires integrating digital monitoring into a structured clinical framework, as technology cannot replace direct supervision. This involves three core components (31). First, comprehensive pre-participation screening is essential to assess clinical stability, functional capacity, and exercise tolerance, identifying patients unsuitable for fully remote protocols (31). Second, adverse event prevention relies on predefined, physiologically based alert thresholds for parameters such as heart rate and oxygen saturation (76). However, remote monitoring has inherent limitations in detecting issues like improper exercise form or subjective distress. Third, clear clinical escalation pathways should be established, specifying protocols for alert triage, clinical decision-making, and emergency referral, alongside providing patients with explicit guidance on seeking urgent care (33). Regular in-person reassessment remains crucial for adjusting prescriptions and confirming safety. Ultimately, remote rehabilitation should operate as a clinician-supervised model augmented by technology, not as an autonomous system. The operational components and minimum structural requirements for safe remote cardiac rehabilitation are summarized in Supplementary Figure S1.

Within this safety governance framework, it is essential to clearly distinguish exercise intensity prescription targets from clinical safety escalation thresholds to avoid conceptual and operational conflation. When a baseline graded exercise test is unavailable, a pragmatic initial aerobic training intensity may be prescribed at 20–30 beats per minute above resting heart rate (RHR + 20–30 bpm), in conjunction with concurrent rating of perceived exertion monitoring (RPE 11–14), as recommended by Mytinger et al. (77). This approach serves as a temporary, convenience-based method for initiating supervised exercise training and should not be interpreted as an indicator of clinical deterioration or as a trigger for escalation. Whenever feasible, formal exercise testing remains the preferred and more precise basis for individualized exercise prescription.

To further enhance safety and standardization in remote exercise-based rehabilitation, adverse events should be explicitly categorized by clinical severity. Minor events include transient symptoms such as dizziness or fatigue that resolve with rest. Moderate events encompass symptomatic hypotension, sustained arrhythmias, or exercise-induced oxygen desaturation below 88% (31, 76). Major events include syncope, chest pain suggestive of myocardial ischemia, ventricular arrhythmias, or hospitalization. In addition to event classification, predefined escalation thresholds should be established to enable timely intervention. Examples include weight gain exceeding 2 kg over three days (12) or exercise-induced desaturation below 88% (76). Supervision intensity should be stratified according to baseline risk profile: low-risk patients may be managed through asynchronous monitoring, moderate-risk patients warrant scheduled weekly reviews, and high-risk patients require hybrid models incorporating in-person oversight. These specifications distinguish structured remote cardiac rehabilitation from generic remote monitoring programs by embedding risk-adapted surveillance and clear safety protocols.

### 4.5 Pragmatic workflow model for remote cardiac rehabilitation

To enhance its clinical applicability, the proposed framework is operationalized into a structured five-step workflow. The process begins with eligibility assessment, which evaluates clinical stability—defined as the absence of recent decompensation—along with frailty, cognitive function, digital literacy, and caregiver support (31, 67). Based on this assessment, patients are assigned to an appropriate program type: low-risk individuals are directed to fully remote monitoring, moderate-risk patients to a hybrid model, and high-risk patients to center-based rehabilitation (32, 34). Once enrolled, a minimum monitoring dataset is collected, including resting and exercise heart rate, weight measured at least three times weekly, symptom scores, and medication adherence (12). Patient data are continuously evaluated through a tiered alert system, which triggers predefined responses: Tier 1 prompts self-management guidance, Tier 2 initiates nurse review within 24 h, Tier 3 requires same-day physician escalation, and Tier 4 mandates emergency referral (31, 76). Finally, periodic reassessment is conducted through in-person clinical review every four to eight weeks to adjust the care plan as needed (31, 32). This stepwise model ensures consistent, risk-stratified delivery of remote cardiac rehabilitation while maintaining patient safety and clinical oversight.

## 5 Future research directions: building a next-generation intelligent cardiac rehabilitation ecosystem

Future research should move beyond validating isolated technologies or short-term outcomes to fully realize the potential of digital health in CHF cardiac rehabilitation. Instead, the focus should shift to building an intelligent, adaptive, and equitable next-generation rehabilitation ecosystem. Achieving this goal requires coordinated innovation across three domains: research paradigms, technological integration, and systemic implementation.

### 5.1 Innovation in research paradigms: towards real-world evidence and comprehensive value assessment

Future efficacy research should transition from strictly controlled trials to more broadly applicable pragmatic clinical studies. Methodologies like pragmatic trials and stepped-wedge cluster designs can evaluate interventions in diverse real-world settings and patient populations, generating evidence more applicable to widespread practice (78, 79). Concurrently, research evaluation should expand beyond clinical endpoints to include comprehensive value assessment. This requires patient-centered, lifetime health economic evaluations that assess direct medical costs and quantify impacts on patient productivity, caregiver burden, and long-term quality of life (80). Such evaluations will provide a robust evidence base for developing value-based payment models and informing policy (81).

### 5.2 Frontiers in technological integration: from automation to intelligence and immersion

Next-generation systems will feature significantly enhanced intelligence. A core objective is exploring fully personalized, adaptive rehabilitation pathways driven by artificial intelligence (82). By continuously learning individual physiological responses, behaviors, and preferences, artificial intelligence systems could dynamically adjust exercise intensity, education, and intervention frequency, achieving true one-to-one optimization. At the monitoring level, integrating advanced biosensors is crucial (83, 84). This includes wearable non-invasive hemodynamic monitors (providing continuous cardiac output and vascular resistance data) and continuous subcutaneous biomarker monitors (e.g., for natriuretic peptides or glucose). These technologies enable more precise assessment of cardiac load and metabolic status. Furthermore, immersive and gamification technologies offer novel ways to enhance patient engagement (85). Immersive virtual environments or theory-based interactive games can transform routine exercises into engaging experiences, potentially improving long-term adherence.

### 5.3 Achieving equity and scalability: focusing on accessibility and systemic support

Technological advancement should promote equity. Research should proactively explore strategies to reduce barriers to technology access and use. Examples include designing devices with simplified interfaces or leveraging widely available technologies like smartphones for multi-channel delivery, ensuring benefits reach diverse socioeconomic and age groups (86, 87). At the systemic level, a key task is advocating for policies that establish value-based payment models for digital cardiac rehabilitation (88). This requires collaboration among researchers, payers, and regulators to define efficacy endpoints, safety standards, and corresponding reimbursement mechanisms. Ultimately, establishing a cross-disciplinary network encompassing clinical medicine, rehabilitation, engineering, data science, public health, and policy is fundamental for translating technology into scalable, sustainable services (87). Through such comprehensive efforts, visionary concepts can be translated into equitable clinical reality.

## 6 Summary

In summary, integrating digital health and remote monitoring technologies represents a potentially transformative but context-dependent approach to the persistent challenges of access and long-term sustainability in cardiac rehabilitation for CHF. This framework moves beyond simply replacing traditional models. It delivers core value through a data-driven, closed-loop system that enables personalized, proactive, and continuous care.

Its ultimate success and broad adoption depend on more than technological maturity. Its sustainability rests on three pillars: (1) generating high-quality real-world evidence focused on long-term outcomes and cost-effectiveness; (2) implementing adaptive changes in healthcare systems, including workflow redesign, role transformation, and payment reform; and (3) a fundamental commitment to health equity, ensuring through intentional design that technological benefits reach all patient groups.

Therefore, we call for cross-disciplinary collaboration among researchers, clinicians, policymakers, and technology innovators. This concerted action is essential for overcoming existing technological and systemic barriers. Only through sustained collaboration can digitally-enabled cardiac rehabilitation evolve from a “promising pilot” into an “accessible, affordable, and efficient routine practice.” This evolution is crucial for improving long-term health outcomes and quality of life for the large population living with CHF.

## Data Availability

The original contributions presented in the study are included in the article/Supplementary Material, further inquiries can be directed to the corresponding author.
