# Supplementary material for: Integrating digital health and remote monitoring: emerging trends in cardiac rehabilitation research for chronic heart failure
Source: Front Cardiovasc Med. 2026 Apr 9;13:1774181. doi: 10.3389/fcvm.2026.1774181 (PMC13102784; doi:10.3389/fcvm.2026.1774181)
Supplement: Supplementary file 1 [file Table1.docx]

**Supplementary Table 1. Mapping Digital Cardiac Rehabilitation Components to Clinical Outcomes**

| **Intervention Ingredient** | **Intensity/Feature** | **Target Population Phase** | **Reported Associated**  **Outcomes** |
| --- | --- | --- | --- |
| Exercise personalization | Real-time HR-guided adjustment | Stable outpatient | ↑Exercise capacity |
| Human coaching  frequency | Weekly structured  contact | Vulnerable post-  discharge | ↑Adherence,  ↓ readmission |
| Monitoring modality | Multi-parameter (HR +weight + PROs) | High-risk | Earlier decompensation  detection |
| Alert design | Tiered+clinician triage | Moderate-high risk | ↓Alert fatigue |
| Program type | Hybrid | Frail/multimorbid | Improved safety profile |
| Follow-up duration  >6 months | Sustained intervention | Stable | Improved QoL persistence |

**Abbreviations**: HR, Heart Rate; PROs, Patient-Reported Outcomes; QoL, Quality of Life.
