# Supplementary figures and images for: Integrating digital health and remote monitoring: emerging trends in cardiac rehabilitation research for chronic heart failure
Source: Front Cardiovasc Med. 2026 Apr 9;13:1774181. doi: 10.3389/fcvm.2026.1774181 (PMC13102784; doi:10.3389/fcvm.2026.1774181)

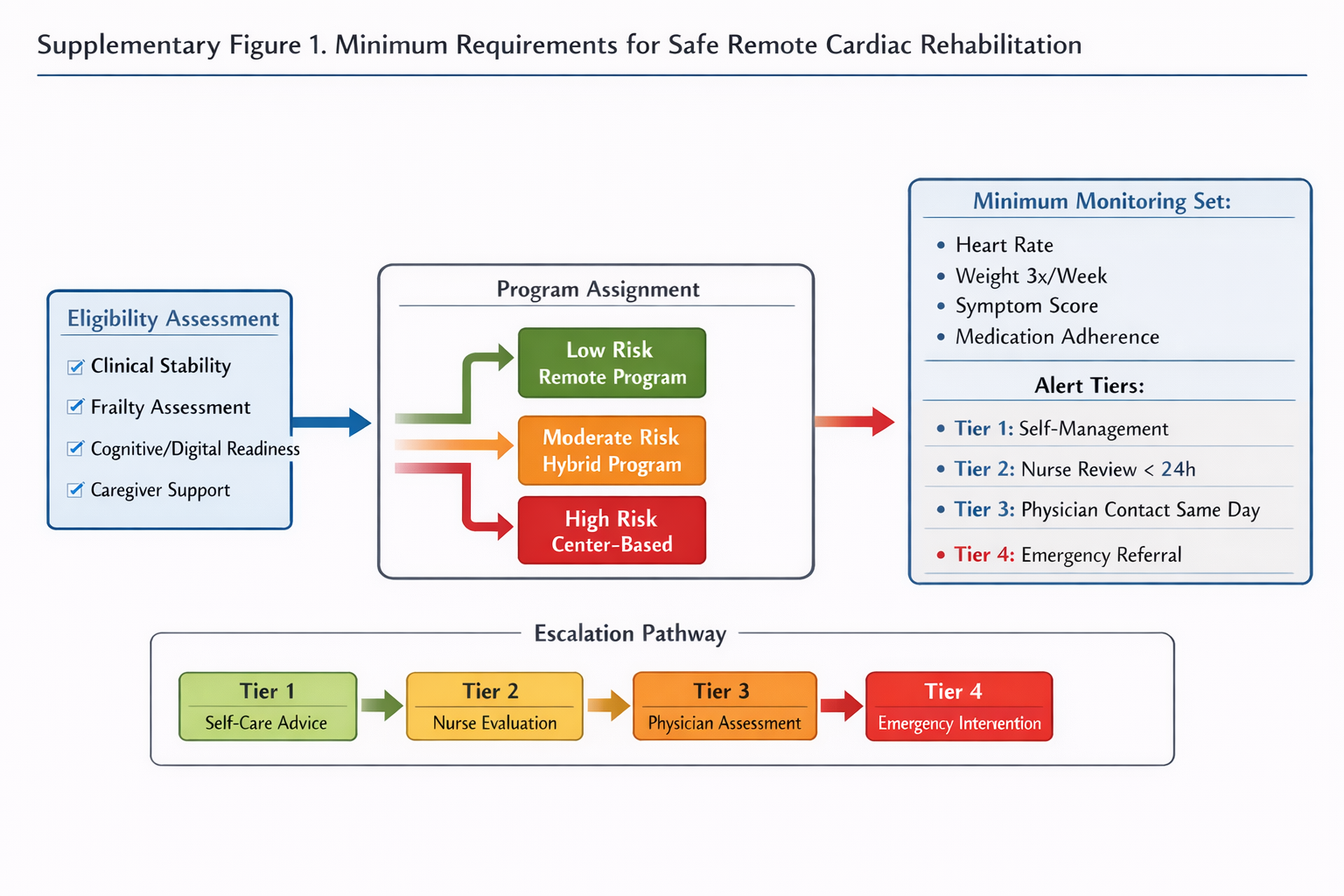

Supplement: Supplementary file 2 [file Image1.tiff]
